# Comparison of As, Cd, Cu, Fe, and Pb levels in dry cat foods containing different protein sources (poultry, fish, red meat) and in cats consuming these diets

**DOI:** 10.1007/s11259-026-11209-0

**Published:** 2026-04-14

**Authors:** Bengü Bilgiç, Duygu Tarhan, Nural Pastacı Özsobacı, Fatma Ateş, Sema Sandıkçı Altunatmaz, Banu Dokuzeylül, Alev Meltem Ercan, Mehmet Erman Or

**Affiliations:** 1 Department of Internal Medicine, Faculty of Veterinary Medicine, Istanbul University- Cerrahpasa, Istanbul, 34320 Türkiye; 2 Department of Biophysics, School of Medicine, Bahcesehir University, Istanbul, 34734 Türkiye; 3 Department of Biophysics, Faculty of Medicine, Istanbul University-Cerrahpasa, Istanbul, 34098 Türkiye; 4 Department of Biophysics, School of Medicine, Bezmialem Vakif University, Istanbul, 34093 Türkiye; 5 Department of Food Processing and Food Technology, Vocational School of Veterinary Medicine, Istanbul University-Cerrahpasa, Istanbul, 34320 Türkiye

**Keywords:** Diet, Heavy metal, Cat foods, Poultry, Fish, Red meat

## Abstract

Heavy metals are an important source of environmental contamination and can enter the pet food chain through animal-derived protein ingredients and drinking water. Owing to their obligate carnivorous nature and strong reliance on animal-based proteins, cats may be exposed to diet-related potentially toxic metals. It was aimed to compare arsenic, cadmium, copper, iron, and lead concentrations in commercial dry cat foods (*n* = 300) formulated with different primary protein sources (poultry, fish, and red meat), and to investigate potential toxic effects in relation to protein source and duration of dietary exposure. Cats (*n* = 750) were grouped according to dietary protein source and feeding duration. Heavy metal concentrations were determined in cat foods, household drinking water, serum, and hair samples using ICP-OES. Cadmium concentrations were significantly higher in fish- and red meat–based diets compared with poultry-based diets; copper and iron concentrations were higher in poultry-based diets. All measured metal concentrations remained below established regulatory limits in diets. In biological samples, no consistent accumulation was observed according to protein source or feeding duration. Cats fed poultry-based diets exhibited higher blood copper and iron concentrations and lower blood lead levels compared with other groups; however, all values remained within physiological reference ranges. Hair metal concentrations did not differ significantly among dietary groups. Heavy metal concentrations measured in the evaluated diets were generally within suggested guidelines. Although copper concentrations exceeded recommended limits by FEDIAF in some diets, this wasn’t associated with clinically relevant accumulation in cats. Given the observational and cross-sectional nature of the study, these findings should be interpreted cautiously and do not exclude the possibility of long-term accumulation or risk associated with chronic exposure.

## Introduction

Heavy metals represent an important group of environmental contaminants within the food chain, and their accumulation in biological systems may result in severe oxidative damage through the excessive generation of superoxide radicals (O₂⁻), hydrogen peroxide (H₂O₂), and hydroxyl radicals (OH⁻). Chronic exposure to toxic concentrations has been associated with mutagenic, carcinogenic, teratogenic, immunosuppressive, nephrotoxic, neurologic, and cardiovascular disorders, as well as impaired growth, body condition, and reproductive performance in both humans and animals (Govind and Madhuri 2014; Meertens et al. 2005; Cheng et al. 2014; Bampidis et al. 2013; Albretsen et al. 2006; Knight et al. 2003). Many heavy metals are absorbed and bioaccumulated by plants and subsequently transferred through terrestrial and aquatic food chains, thereby affecting a wide range of animal species in addition to humans (Mudgal et al. 2010; Zhuzzhassarova et al. 2024).

Cats are obligate carnivores due to their specific metabolic characteristics, including an essential dietary requirement for taurine and the inability to convert carotenoids to retinol. Consequently, animal-based proteins constitute the major component of their diet. Compared with dogs, cats require relatively higher dietary protein levels. This requirement is not primarily related to lower protein digestibility but rather to metabolic adaptations associated with their carnivorous diet, including the limited ability to down-regulate hepatic enzymes involved in protein catabolism when dietary protein intake decreases (Kendall et al. 1982; Morris and Rogers 1982; Morris 2002). Commercial cat foods commonly contain protein sources derived from poultry, ruminant meat, and various fish species. However, heavy metal contamination has been reported in poultry, livestock, and aquatic organisms, particularly in regions affected by environmental pollution, and these contaminants may accumulate in edible tissues and enter the pet food production chain (Xiong et al. 2010; Wang et al. 2013; Akan et al. 2010; Zhuang et al. 2009; Hussain et al. 2012; Hu et al. 2018; Lane et al. 2015; Abdel Basset et al. 2014; Or 2005). Several investigations have reported detectable or elevated concentrations of heavy metals in various commercial pet foods and in biological samples such as blood and hair of companion animals consuming these diets, suggesting the potential for chronic dietary exposure (Rzymski et al. 2015; Skibniewska et al. 2017; Badea et al. 2019).

Given that cats are strictly carnivorous and highly dependent on animal-derived protein sources, they may represent a sensitive indicator species for dietary exposure to heavy metals originating from different protein ingredients. In particular, comparative data evaluating poultry-, red meat-, and fish-based diets in relation to metal concentrations in feline biological samples remain scarce, especially in Türkiye. We hypothesized that heavy metal concentrations in commercial dry cat foods differ according to the predominant protein source and that dietary exposure may be reflected in biological matrices such as blood and hair. In this context, the present study aimed to compare the concentrations of As, Cu, Pb, Cd, and Fe in dry cat foods formulated with three major protein sources (poultry, red meat, and fish) and to determine the levels of these metals in cats consuming these diets in Türkiye. Furthermore, correlations between metal concentrations in diets and biological samples were evaluated, with the objective of assessing potential long-term exposure risks.

## Materials and methods

### Diet samples

In this study, 300 samples of premium commercial cat foods from different brands, formulations, and flavors commonly used in feline nutrition were included. All pet food samples were obtained from the manufacturers in their original, unopened packaging and were stored under appropriate conditions until analysis, considering their expiration dates. The commercial dry foods, formulated as both prescription and non-prescription diets, were produced in the USA, Italy, France, Spain, and Türkiye.

The primary protein source of each commercial diet was determined according to the ingredient composition declared by the manufacturer on the product label. No independent quantitative assessment of protein composition was performed. The primary protein sources included in the study consisted of dehydrated poultry-derived proteins (chicken and turkey), hydrolyzed red meat proteins (pork, beef, and lamb), dehydrated and hydrolyzed fish-derived proteins, as well as various plant-based proteins. Accordingly, the pet foods included in the study were classified based on their protein sources as follows:

- Group A (*n* = 100): Foods containing poultry-derived proteins as the primary protein source,
- Group B (*n* = 100): Foods containing fish-derived proteins as the primary protein source,
- Group C (*n* = 100): Foods containing red meat were used as the primary protein source,

In addition, 171 water samples corresponding to the household drinking water consumed by animals for a minimum of 3 months were also collected and analyzed.

## Animal samples

A total of 750 cats from different breeds, ages, and sexes were included in the study and were grouped according to the protein source of the diet they were fed, with 250 cats assigned to each group. Only cats that had been consuming the same dry food for at least three months were enrolled, and those receiving diets containing mixed or multiple protein sources and vitamin/mineral supplements were excluded from the study. In the study, only cats presented to the Veterinary Internal Medicine Clinic were evaluated, and all animals were privately owned indoor cats with no contact with other animals or the external environment. Information regarding the cats’ feeding practices and the type of food provided was obtained from the owners using a standardized questionnaire. Cats fed red-meat-based, fish-based, or poultry-based protein diets were further subdivided according to the duration of feeding with the respective diet into the following subgroups:

- Cats fed a poultry-derived diet for at least 3 months (Group A3, *n* = 77), at least 6 months (Group A6, *n* = 61), at least 12 months (Group A12, *n* = 45), and at least 24 months (Group A24, *n* = 67).
- Cats fed a fish-derived diet for at least 3 months (Group B3, *n* = 67), at least 6 months (Group B6, *n* = 56), at least 12 months (Group B12, *n* = 58), and at least 24 months (Group B24, *n* = 69).
- Cats fed a red meat-based diet for at least 3 months (Group C3, *n* = 65), at least 6 months (Group C6, *n* = 60), at least 12 months (Group C12, *n* = 62), and at least 24 months (Group C24, *n* = 63).

Blood samples (5 mL in total) were collected from the jugular vein of each cat. Of this volume, 2 mL was transferred into EDTA-containing tubes, mixed with an equal volume of 20% trichloroacetic acid (TCA), and centrifuged; the supernatant was then separated. The remaining 3 mL of blood was placed into anticoagulant-free tubes and centrifuged at 3,000–5,000 rpm to obtain serum samples. In addition to blood sampling, hair samples from the dorsal cervical region were also collected from each cat. All hair, serum, and plasma samples were stored at − 80 °C in a deep freezer until the day of analysis.

## Heavy metal measurements

Arsenic (As), cadmium (Cd), copper (Cu), iron (Fe), and lead (Pb) concentrations were determined by an inductively coupled plasma-optical emission spectrophotometer (ICP-OES; Thermo iCAP 6000series). Diet, water, and hair samples were weighed in pre-tarred, heat-resistant graduated glass tubes, and their quantities were determined before digestion. The samples were prepared for elemental analysis using the wet digestion method. Trace element analysis was performed using the ICP-OES methodology as previously described in our published studies (Bilgiç et al., 2026; Katsoulos et al. 2025; Draghi et al. 2024; Agradi et al. 2023).

For wet digestion, 1 mL of 55–65% nitric acid (HNO₃) was added to the feed and hair samples, which were then placed in a laboratory drying oven (Heraeus W.C., Hanau, Germany) at 100–120 °C for approximately one hour to allow dissolution. After removal from the oven and cooling to room temperature, 1 mL of 65% perchloric acid (HClO₄) was added, and the samples were incubated for an additional hour at 100–120 °C to complete the wet digestion procedure. Following digestion, the samples were cooled to room temperature and diluted with distilled water, which was also used as the blank solution in ICP-OES analysis and for the preparation of calibration standards. The solutions were mixed using a vortex mixer, and the final dilutions were prepared before elemental measurement. Element concentrations obtained from ICP-OES were calculated using the formula: (Element concentration × dilution factor) / sample weight. The results were expressed as µg/g sample. For each feed sample, subsamples were collected from three different portions of the product; these were digested separately, and their mean value was used as the final elemental concentration in order to obtain more reliable measurements in feed and water samples.

Serum samples were diluted 1:10 with distilled water before the determination of As, Cd, Cu, Fe, and Pb, and the results were expressed as µg/mL.

The concentrations of As, Cd, Cu, Fe, and Pb in the samples were determined using ICP-OES at the wavelengths appropriate for each element, as presented in Table 1. ICP-OES measurements were carried out using the following instrumental parameters: plasma gas flow rate of 15 L/min, argon carrier flow rate of 0.5 L/min, sample flow rate of 1.51 L/min, peristaltic pump speed of 100 rpm, and RF power of 1150 W. The element-specific limits of detection (LoD) for liquid samples were 0.001 µg/mL for As, 0.001 µg/mL for Cd, 0.012 µg/mL for Cu, 0.011 µg/mL for Fe, and 0.002 µg/mL for Pb. For solid samples, the element-specific LoDs were expressed in µg/g. The recovery of the analyzed quality control was between 94% and 108%.

**Table 1:** ICP-OES wavelengths for As, Cd, Cu, Fe, and Pb analysis

Elements	Wavelengths (nm)
As	189.042
Cd	228.802
Cu	324.754
Fe	259.940
Pb	220.353

### Statistical Analysis

Statistical analyses were performed using IBM SPSS Statistics version 25.0 (IBM Corp., Armonk, NY, USA). The distribution of continuous variables was assessed using the Shapiro–Wilk test. As the variables were not normally distributed, comparisons among groups were conducted using the Kruskal–Wallis H test. When significant differences were detected, pairwise comparisons were performed using the Dunn–Bonferroni post hoc test to control for the increased risk of Type I error associated with multiple comparisons. Spearman’s rank correlation test was used for correlation analyses. A p-value of < 0.05 was considered significant.

## Results

The age range, body weight, and sex of the cats are presented in Table 2. Heavy metal concentrations in poultry-based, fish-based, and red-meat-based cat foods are presented in Table 3, whereas those measured in water samples are presented in Table 4.

**Table 2:** The age range, body weight, and sex of the cats

Group	Age Range	Body Weight (kg)	Females (*n*)	Males (*n*)
A3	4 months–10 years	2–7	45	32
A6	7 months–13 years	2.4–7.3	32	29
A12	1–9 years	2–8.7	18	27
A24	1–14 years	2.7–9	29	38
B3	6 months–9 years	2.5–8	34	33
B6	8 months–8 years	3.2–5	27	29
B12	1–8 years	3–6	27	31
B24	2–15 years	2.9–8.5	34	35
C3	6 months–8 years	3–5	33	32
C6	1–8 years	3–4.5	30	30
C12	3–8 years	3–4.5	30	32
C24	2.5–15 years	2.6–6	30	33

**Table 3:** Heavy metal concentrations in poultry-based, fish-based, and red-meat-based cat foods

Groups		As (µg/g)	Cd (µg/g)	Cu (µg/g)	Fe (µg/g)	Pb (µg/g)
Group A (*n* = 100)	Mean	0.77	0.10^a^	24.6^a^	192.3^a^	0.35^a^
	Min-Max	0.04–3.68	0.01–0.86	4.85–120.7	45.0-7505	0.003–1.61
	SD	0.60	0.07	13.19	583.2	0.34
Group B (*n* = 100)	Mean	1.01	0.16^b^	25.2^a^	150.7^a^	0.44^ab^
	Min-Max	0.001–2.86	0.01–1.26	2.83–75.14	54.2-302.6	0.03–1.61
	SD	0.77	0.17	14.0	55.5	0.43
Group C (*n* = 100)	Mean	1.84	0.36^b^	12.1^b^	88.9^b^	0.59^b^
	Min-Max	0.001–7.87	0.091–1.68	0.38–32.8	63.8-160.8	0.21–1.26
	SD	2.46	0.42	12.0	31.0	0.38
MTL (µg/g)		10* 12.5**	2* 10**	28***	681.8***	5* 10**

MTL: Maximum tolerable limits

^a, b^: Different letters within the same column indicate significant differences

* Reference value for complete feed for pet animals, according to the European Union (EU 2002)

** Reference value according to the United States Food and Drug Administration (FDA, 2011)

*** Reference value according to the FEDIAF (FEDIAF 2025)

**Table 4:** Heavy metal concentrations in water samples

Mean	As (µg/ml)	Cd (µg/ml)	Cu (µg/ml)	Fe (µg/ml)	Pb (µg/ml)
	0.016	0.001	0.019	0.028	0.006
**n** ^*^	160	82	171	65	111
**SD**	0.025	0.004	0.048	0.097	0.008

^*^Among the 171 analyzed water samples, some measurements for As, Cd, Fe, and Pb were below the analytical detection limit and therefore could not be quantified

The mean Cd levels were significantly higher in the Group B and the Group C compared with the Group A. Conversely, the mean Cu and Fe concentrations were significantly higher in Groups A and B than in Group C. The mean Pb concentrations were significantly lower in Group C compared with Group A. No significant differences were observed among diet groups with respect to As concentrations.

The findings regarding the comparison of blood heavy metal levels across dietary protein sources are presented in Table 5. Accordingly, no significant differences were detected among the Group A, Group B, and Group C subgroups in the comparisons performed according to feeding duration within each protein source category.

**Table 5:** Comparison of blood heavy metal levels according to the dietary protein source

Groups	As (µg/ml)		Cd (µg/ml)		Cu (µg/ml)			Fe (µg/ml)		Pb (µg/ml)	
	Mean ± SD	Median (IQR)	Mean ± SD	Median (IQR)	Mean ± SD		Median (IQR)	Mean ± SD	Median (IQR)	Mean ± SD	Median (IQR)
Group A3 (*n* = 77)	0.22 ± 0.10	0.23 (0.16–0.29)	0.79 ± 0.62	0.82 (0.30–1.01)	1.38^a^±0.63	1.27 (0.92–1.67)		2.74^a^±1.95	2.20 (1.48–3.48)	0.61^a^±0.50	0.49 (0.24–0.79)
Group B3 (*n* = 67)	0.23 ± 0.15	0.24 (0.08–0.28)	0.68 ± 0.61	0.41 (0.31–0.89)	0.68^b^±0.48	0.52 (0.27–1.02)		1.37^b^±0.60	1.35 (0.91–1.55)	2.23^b^±1.45	1.70 (1.50–2.26)
Group C3 (*n* = 65**)**	0.21 ± 0.10	0.26 (0.13–0.29)	0.95 ± 0.61	1.02 (0.30–1.41)	0.88^ab^±0.63	0.92 (0.29–1.40)		1.30^b^±0.71	1.13 (0.92–1.49)	3.36^b^±2.96	1.74 (1.35–6.33)
*p*	>0.05		> 0.05		**<0.001**			**<0.001**		**<0.001**	
Group A6 (*n* = 61)	0.23 ± 0.12	0.23 (0.19–0.29)	0.75 ± 0.71	0.52 (0.30–0.96)	1.41^a^±0.64	1.39 (0.97–1.73)		2.28^a^±1.17	2.02 (1.64–2.64)	0.78^a^±0.64	0.73 (0.30–0.96)
Group B6 (*n* = 56)	0.17 ± 0.10	0.16 (0.09–0.20)	0.36 ± 0.24	0.31 (0.22–0.48)	0.59^b^±0.34	0.46 (0.40–0.82)		1.15^b^±0.43	1.12 (0.91–1.43)	1.89^b^±0.66	1.70 (1.31–2.53)
Group C6 (*n* = 60)	0.25 ± 0.13	0.23 (0.17–0.28)	0.42 ± 0.35	0.31 (0.23–0.34)	0.39^b^±0.33	0.20 (0.16–0.64)		1.29^b^±0.60	1.34 (1.02–1.80)	2.49^b^±0.81	2.50 (1.90–3.09)
*p*	>0.05		> 0.05		**<0.001**			**<0.001**		**<0.001**	
Group A12 (*n* = 45)	0.24 ± 0.07	0.25 (0.19–0.28)	0.58 ± 0.43	0.31 (0.30–0.86)	1.62^b^±0.65	1.53 (1.12–2.04)		2.64 ± 1.68	2.21 (1.44–3.47)	0.53^a^±0.54	0.30 (0.18–0.85)
Group B12 (*n* = 58)	0.26 ± 0.13	0.23 (0.20–0.36)	0.67 ± 0.54	0.32 (0.29–0.95)	0.63^a^±0.44	0.51 (0.28–0.93)		1.61 ± 0.69	1.47 (1.06–1.99)	2.18^b^±1.18	1.88 (1.23–3.10)
Group C12 (*n* = 62)	0.18 ± 0.06	0.18 (0.15–0.24)	0.79 ± 0.25	0.86 (0.72–0.97)	1.64^b^±0.64	1.59 (1.09–2.31)		2.01 ± 0.49	2.06 (1.59–2.43)	1.01^ab^±0.20	0.89 (0.83–1.23)
*p*	>0.05		> 0.05		**<0.001**			>0.05		**<0.001**	
Group A24 (*n* = 67)	0.20 ± 0.10	0.20 (0.13–0.27)	0.70 ± 0.54	0.48 (0.29–0.95)	1.38^a^±0.68	1.13 (0.88–1.77)		2.04^a^±1.10	1.77 (1.17–2.72)	0.68^a^±0.58	0.50 (0.29–1.02)
Group B24 (*n* = 69)	0.22 ± 0.11	0.23 (0.12–0.28)	0.60 ± 0.41	0.35 (0.23–0.96)	0.58^b^±0.48	0.41 (0.20–0.79)		1.28^b^±0.67	1.22 (0.80–1.58)	2.25^b^±1.27	1.97 (1.35–2.77)
Group C24 (*n* = 63)	0.22 ± 0.07	0.23 (0.15–0.25)	0.80 ± 0.53	0.82 (0.29–1.17)	0.64^b^±0.31	0.72 (0.33–0.92)		2.01^ab^±1.08	1.48 (1.33–3.32)	2.21^a^±3.15	0.64 (0.17–3.09)
*p*	>0.05		> 0.05		**<0.001**			**0.002**		**< 0.001**	

^a, b^: Different letters within the same column indicate significant differences

In the comparisons of groups that were fed similar diets for at least 3 months, the mean Cu concentration in Group A3 was found to be significantly higher than that of Group B3. Similarly, the mean Fe in Group A3 was significantly higher than in Groups B3 and C3, whereas the mean Pb concentration in Group A3 was significantly lower compared with the other groups. The mean Cu in Group A6 was significantly higher than in Group B6 and Group C6, and the mean Fe concentration in Group A6 was significantly higher than in Group B6 and Group C6. The mean Pb concentration in Group A6 was also significantly lower than in the other groups. The mean Cu in Group B12 was lower than in Group A12 and Group C12. In contrast, the mean Pb concentration in Group B12 was higher than in Group A12. The mean Cu in Group A24 was significantly higher than in Group B24 and Group C24. The mean Fe concentration in Group A24 was also higher than in Group B24. Conversely, the mean Pb concentration in Group B24 was significantly higher than in Group A24 and Group C24 (Table 5).

The results of heavy metal measurements performed on hair samples obtained from cats are presented in Table 6. Accordingly, in the analyses of As, Cd, Cu, Fe, and Pb concentrations measured from the hair samples of cats that had been fed the same protein source — poultry-based, fish-based, or red-meat-based diets — for at least 3, 6, 12, and 24 months, no significant differences were detected among the groups.

**Table 6:** Comparison of heavy metal levels in hair samples according to the dietary protein source

Groups	As (µg/g)		Cd (µg/g)		Cu (µg/g)			Fe (µg/g)		Pb (µg/g)	
	Mean ± SD	Median (IQR)	Mean ± SD	Median (IQR)	Mean ± SD		Median (IQR)	Mean ± SD	Median (IQR)	Mean ± SD	Median (IQR)
Group A3 (*n* = 77)	2.67 ± 1.90	2.36 (1.31–3.47)	0.49 ± 0.73	0.22 (0.10–0.45)	10.9 ± 4.15	11.40 (7.85–13.5)		173 ± 126	153.4 (62.3-281.9)	2.33 ± 2.19	1.19 (0.90–3.65)
Group B3 (*n* = 67)	4.67 ± 2.92	5.01 (2.94–5.79)	0.39 ± 0.36	0.24 (0.13–0.67)	8.84 ± 3.89	8.16 (5.47–11.1)		131 ± 102	95.7 (46.1-211.2)	4.17 ± 3.53	3.26 (0.86–7.57)
Group C3 (*n* = 65)	2.57 ± 0.16	2.67 (2.38–2.70)	0.13 ± 0.14	0.07 (0.02–0.34)	7.68 ± 1.21	8.03 (6.68–8.93)		173 ± 210	105.5 (41.6-229.6)	3.05 ± 3.56	1.40 (0.14–8.14)
***p***	> 0.05		> 0.05		> 0.05			> 0.05		> 0.05	
Group A6 (*n* = 61)	3.38 ± 1.90	3.18 (1.52–5.01)	0.30 ± 0.19	0.29 (0.13–0.44)	9.12 ± 3.09	9.35 (7.20–11.4)		134 ± 123	86.26 (59.5-180.9)	2.82 ± 3.33	1.42 (0.99–2.86)
Group B6 (*n* = 56)	2.84 ± 2.41	1.97 (1.10–5.08)	0.28 ± 0.25	0.26 (0.02–0.51)	9.75 ± 3.62	9.06 (6.24–13.6)		164 ± 177	69.3 (54.2–215)	1.42 ± 0.90	1.46 (0.76–1.83)
Group C6 (*n* = 60)	2.59 ± 0.14	2.67 (2.38–2.70)	0.14 ± 0.14	0.07 (0.05–0.34)	8.32 ± 0.87	8.48 (7.76–9.01)		233 ± 231	111.2 (89.7-567.5)	3.05 ± 3.56	1.40 (0.14–8.14)
***p***	> 0.05		> 0.05		> 0.05			> 0.05		> 0.05	
Group A12 (*n* = 45)	3.38 ± 4.16	2.14 (0.89–7.77)	0.12 ± 0.09	0.09 (0.04–0.21)	10.1 ± 4.24	10.2 (6.76–12.2)		78.1 ± 43.4	64.3 (43.4–124)	2.79 ± 2.61	1.66 (1.28–6.23)
Group B12 (*n* = 58)	4.58 ± 4.05	2.47 (1.36–7.88)	0.22 ± 0.24	0.19 (0-0.39)	7.58 ± 3.62	6.31 (5.62–8.25)		61.5 ± 79.6	52.2 (48.8–84.7)	4.67 ± 4.35	3.30 (1.79–6.88)
Group C12 (*n* = 62)	2.56 ± 0.15	2.67 (2.38–2.70)	0.15 ± 0.13	0.08 (0.07–0.34)	7.86 ± 0.87	8.03 (6.94–8.93)		218 ± 242	105.5 (42.1-567.5)	3.72 ± 3.84	1.39 (0.14–8.14)
***p***	> 0.05		> 0.05		> 0.05			> 0.05		> 0.05	
Group A24 (*n* = 67)	3.29 ± 2.33	3.29 (1.14–5.43)	0.24 ± 0.34	0.16 (0-0.28)	10.6 ± 3.61	9.66 (7.84–13.3)		183 ± 126	157 (82.9–255)	4.55 ± 3.14	4.23 (1.58–6.79)
Group B24 (*n* = 69)	0.85 ± 0.39	0.67 (0.61–1.05)	0.22 ± 0.19	0.11 (0.07–0.43)	8.98 ± 2.27	9.10 (7.00-10.4)		172 ± 64.3	179 (110–198)	1.67 ± 1.13	1.35 (0.86–3.29)
Group C24 (*n* = 63)	2.63 ± 0.13	2.69 (2.61–2.71)	0.12 ± 0.11	0.07 (0.06–0.14)	8.35 ± 0.82	8.93 (7.76–9.1)		370 ± 255	567 (89.7–582)	5.19 ± 3.83	8.14 (1.08–8.21)
***p***	>0.05		> 0.05		>0.05			> 0.05		> 0.05	

**Fig. 1:**
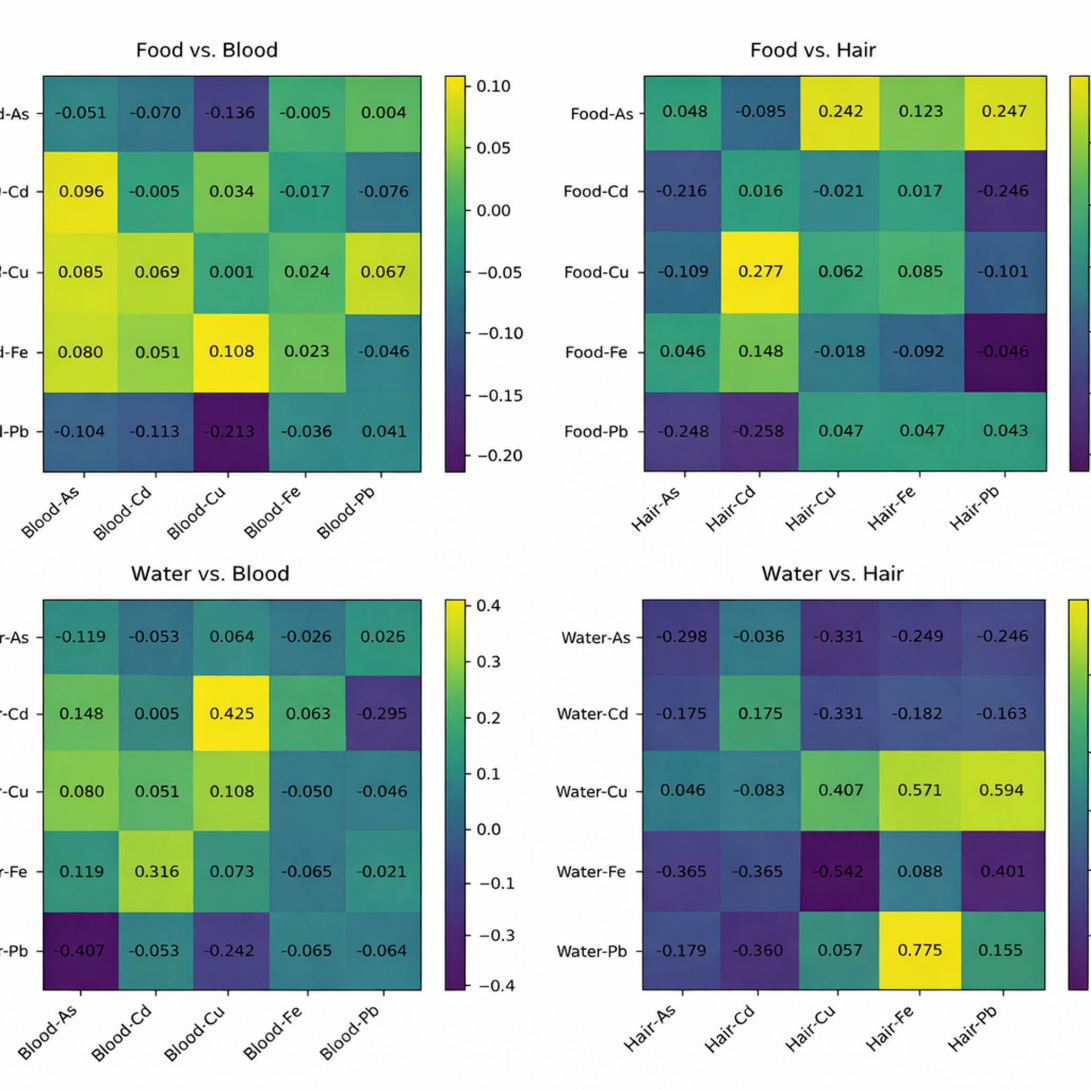
Heat maps illustrating the correlations between food–blood, food–hair, water–blood, and water–hair heavy metal concentrations

In the correlation analyses performed between blood heavy metal levels and those measured in cat food and hair samples, a significant association was observed between food Pb and blood Cu (*p* = 0.031, *r* = − 0.213), and between food Fe and hair Pb (*p* = 0.031, *r* = − 0.360). Similarly, in the correlation analyses between water and blood heavy metal levels, significant relationships were detected between blood As and water Pb (*p* = 0.002, *r* = − 0.407) and between blood Cu and water Cd (*p* = 0.007, *r* = 0.425). A significant correlation was found between water Fe and hair Cu (*p* = 0.037, *r* = − 0.542) in the analyses of water and hair heavy metal levels (Fig. 1).

## Discussion

### Arsenic

As arsenic is regarded as an undesirable toxic element, no specific maximum limit has been established by AAFCO, NRC, or FEDIAF. However, the U.S. Food and Drug Administration has reported a legally tolerable limit of 12.5 µg/g for arsenic in pet foods (FDA 2011). According to EU Directive 2002/32/EC and Commission Regulation (EU) 2019/1869, the maximum permitted level of arsenic in complete feed is 2 µg/g, although a higher limit of 10 µg/g is allowed for pet foods containing fish or other aquatic products (EU 2002). In a comparable study evaluating arsenic levels in cat foods according to protein source, the mean arsenic concentration in fish-based, red meat–based, and poultry-based cat diets was reported as 0.000 mg/kg (Zafalon et al. 2021). In contrast, another study reported a wide range of arsenic concentrations in fish-based cat diets, with minimum and maximum values of 0.86–12.50 µg/g (Squadrone et al. 2017). In a more recent comparable study, the mean arsenic concentrations measured in commercial cat foods were reported as 2.77 µg/g (Bilgiç et al. 2026). In a previous study on commercial dog foods in USA, higher levels of arsenic, cadmium, and mercury were detected in fish-based diets compared to poultry or red meat-based diets (Kim et al. 2018). However, in this study, no significant differences were observed in arsenic concentration among all food groups. Moreover, the arsenic levels were consistent with the previous reports and remained below the maximum limit established by the U.S. Food and Drug Administration. According to EU recommendations, the arsenic limit for human consumption in drinking water is 0.01 µg/mL (EU 2020). In the current study, the mean arsenic concentration is slightly higher than the recommended limit; however, the results should be interpreted with caution, as guideline values are established for drinking water intended for human consumption.

No significant differences in arsenic concentrations were observed in blood or hair samples of cats consuming a specific protein source when evaluated according to the feeding duration. In an effort to define acceptable reference values for arsenic exposure in cats, Buchweitz et al. (2019) reported whole-blood arsenic concentrations ranging from 0.022 to 2.56 µg/mL in domestic cats. Blood arsenic concentrations observed in the present study were within this previously reported range. A study evaluating arsenic concentrations in feline hair samples using inductively coupled plasma mass spectrometry reported a mean hair arsenic level of 137.63 µg/g in the healthy cats (Badea et al. 2024). The marked discrepancy between this reported value and the findings of the present study appears to primarily reflect differences in the analytical methodologies employed. Overall, arsenic levels in commercial cat foods appear to be independent of whether the primary protein source is aquatic or terrestrial.

### Cadmium

In a study evaluating cadmium levels in dog foods in the USA, fish-based diets were reported to contain higher mean cadmium concentrations than poultry- and red meat–based diets (Kim et al. 2018). In a recent study evaluating the heavy metal contamination in pet foods in China, the mean cadmium concentration was 0.142 µg/g (Du et al. 2025). In another study from Brazil, higher mean cadmium levels in cat foods were reported, with a mean concentration of 2.18 µg/g (Zafalon et al. 2021). According to the FDA, the maximum tolerable cadmium level is 10 µg/g (FDA 2011). Maximum tolerable limit value for complete feed was reported as 2 µg/g for pet animals, according to the European legislation (EU 2002). In the present study, the cadmium concentrations detected in cat foods were consistent with the previous studies and remained below the established reference limits. The highest cadmium concentrations were observed in red meat–based foods. The observed differences may be partially explained by variations in environmental exposure. While poultry are generally fed controlled, industrially formulated diets, farm animals may be directly exposed to environmental cadmium through contaminated soil, water, and forage. In livestock, cadmium exposure has been associated with anthropogenic factors such as industrial activities, mining operations, soil contamination, and agricultural practices. Additionally, in certain regions, naturally cadmium-rich soils and contaminated feedstuffs may further increase exposure risk, potentially leading to accumulation in tissues and hair (Draghi et al. 2023; Castrica et al. 2025). However, when compared with data reported by these nutritional guidelines, the cadmium levels detected in the present study appear to remain within ranges not generally associated with toxicological concern.

According to EU regulations, the legal limit for cadmium in drinking water is 0.005 µg/mL, and cadmium concentrations measured in water samples in the present study were substantially below this threshold (EU 2020). However, it should be noted that this regulatory limit applies to human drinking water standards.

Although studies measuring blood cadmium levels in healthy cats are quite limited, plasma cadmium concentrations in healthy cats measured between 0.0001 and 0.009 µg/mL by using a Perkin-Elmer Model 4110 ZL graphite furnace atomic absorption spectrometer equipped with a transversely heated graphite atomizer (Smetkova et al. 2002). In another study, the mean cadmium concentration in hair samples obtained from healthy cats was reported as 0.15 µg/g by using a Thermo iCAP ICP-OES spectrometer, and the values reported in that study are consistent with those observed in the present study (Badea et al. 2016).

### Copper

According to guidelines published by the NRC, a copper concentration of approximately 5 µg/g on a dry matter basis is considered adequate to meet the dietary requirements of cats (NRC 2006). According to the AAFCO guidelines, the recommended minimum copper requirement for cats during growth and the reproduction period is 15 mg/kg, and for adult maintenance is 5 µg/g (AAFCO 2015). However, a safe upper limit or maximum tolerable intake level for copper in feline diets has not been established by these nutritional guidelines. In contrast, the recent FEDIAF guidelines report a maximum acceptable copper concentration of 2.80 mg per 100 g dry matter (28 µg/g) in commercial cat foods (FEDIAF 2025). According to EU legislation regulating trace element additives in animal feed, the maximum permitted copper content in complete feed varies among animal species. For example, the maximum levels are 15 mg/kg for ovines, 35 mg/kg for caprines, 30 mg/kg for bovines after the start of rumination, and up to 150 mg/kg for piglets, whereas for other animal species, including companion animals such as cats and dogs, the maximum permitted copper level in complete feed is 25 mg/kg (EU 2025).

In the present study, the mean copper concentrations across all diet groups were sufficient to meet the nutritional requirements recommended by AAFCO and NRC and remained below the maximum tolerable limits recommended by FEDIAF. However, according to our results, 18.9% of poultry-based diets, 26.1% of fish-based diets, and 22.2% of red meat–based diets exceeded the copper concentrations recommended by the FEDIAF. Conversely, copper concentrations were below 5 µg/g in 1% of poultry-based, 7% of fish-based, and 27% of red meat–based diets. Similarly, a previous study investigating copper concentrations in commercial cat foods reported that several prescription diets exceeded recommended limits, whereas some non-prescription diets failed to meet minimum nutritional requirements (Bilgiç et al. 2025). In a similar study conducted on dog foods, a considerable proportion of commercial dog foods were reported to be non-compliant with the minimum copper requirements established by FEDIAF (Or et al. 2024).

For human drinking water, the U.S. Environmental Protection Agency has established a copper limit of 1.3 µg/mL, while both the European Union and the World Health Organization have set a guideline value of 2.0 µg/mL (Fitzgerald 1998; EU 2020). Based on the concentrations measured in the present study, the copper concentrations measured in the present study were below these guideline values. Although cats fed poultry-based diets exhibited higher serum copper concentrations, all measured values remained within physiological reference ranges reported by Fascetti et al. (2002). In a study evaluating copper concentrations in the hair of female and male cats kept indoors and outdoors, the mean hair copper levels of indoor-housed female and male cats were reported as 10.5 and 19.4 µg/g, respectively, whereas the corresponding values in outdoor-kept cats were 14.3 and 10.3 µg/g (Goran et al. 2017). Although these findings are generally consistent with the results of the present study, no marked differences between the groups were observed.

### Iron

According to AAFCO recommendations, the minimum iron requirement for cat nutrition is 80 mg/kg dry matter (AAFCO 2015), whereas according to FEDIAF, the upper limit for iron content in adult cat foods is 68.18 mg per 100 g dry matter (681.8 µg/g) (FEDIAF 2025). In the present study, only one poultry-based diet exceeded this upper limit. Nevertheless, the significantly higher mean iron concentrations observed in poultry-based diets compared with red meat–based diets may be associated with the elevated blood iron levels detected in cats fed poultry-based diets for at least three and six months. The composition of commercial diets may vary substantially depending on the inclusion of organ tissues such as liver and the use of trace element supplementation during feed formulation. Therefore, the higher iron concentrations observed in poultry-based diets in the present study may be related to ingredient composition and supplementation practices rather than to the intrinsic iron content of poultry tissue.

The United States Environmental Protection Agency has established a secondary standard value of 0.3 µg/mL for iron in drinking water (EPA 2025). The mean iron concentration measured in drinking water samples in the present study was relatively low and remained below this reference value.

In a study conducted in cats, the serum iron concentration range for healthy cats was reported as 0.30–1.64 µg/mL (Hunt and Jugan 2021), while a similar study reported a range of 0.41–2.08 µg/mL (Gest et al. 2015). Serum iron values obtained in the present study are comparable to those previously reported. In addition, mean iron concentrations measured in hair samples of indoor-kept and free-roaming cats were reported as 40.4 and 74.4 µg/g in females, and 140.4 and 128.2 µg/g in males, respectively (Skibniewska et al. 2011). Although the minimum and maximum iron concentrations observed across all groups in the present study covered a wide range, overall values were consistent with those reported in previous investigations.

### Lead

The U.S. Food and Drug Administration has established a maximum tolerable lead concentration of 10 µg/g for cat and dog foods (FDA 2011). However, by the EU Directive, the maximum tolerable lead concentration in complete feed has been reported as 5 µg/g (EU 2002). In a study comparable to the present investigation, the mean lead concentration measured in commercial cat foods was reported as 9.13 µg/g (Zafalon et al. 2021). In another study investigating heavy metal levels in dog foods according to protein source, mean lead concentrations (0.091 mg/Mcal) in red meat–based diets were significantly higher than those in poultry-based diets (Kim et al. 2018). Consistent with the findings of this study, significantly higher mean lead concentrations in red meat–based cat diets compared with poultry-based diets were obtained. In addition, lead concentrations measured in all food samples remained below the maximum limits specified by the FDA.

Lower blood lead concentrations were observed in cats fed poultry-based diets compared with those consuming red meat– and fish-based diets. This observation highlights the relevance of soil and water-derived lead contamination and exposure within both aquatic and terrestrial food chains. The lower lead concentrations observed in poultry-based diets and in cats consuming these diets may be attributed to the strictly controlled, conventional cage systems of modern poultry production.

Although no universally accepted or standardized reference limits exist for lead concentrations in feline hair samples, and available data remain limited, a study evaluating hair lead levels in household cats reported that mean concentrations ranged from 0.9 to 1.3 µg/g (Skibniewski et al. 2013). Lead concentrations measured in hair samples in the present study were more variable and, in some cases, relatively higher.

According to EU quality standards, the maximum permissible lead concentration in drinking water is 0.005 µg/mL (EU 2020), whereas the WHO recommends an upper limit of 0.01 µg/mL (WHO 2022). Lead contamination in drinking water has been primarily associated with lead-containing plumbing systems and polluted water sources (Jarvis and Fawell 2021). In the present study, mean lead concentrations measured in water samples were slightly higher than EU quality standards, while remaining below WHO health-based guidelines. The variable lead concentrations observed in hair samples may therefore reflect not only environmental exposure but also chronic intake of drinking water contamination.

In the present study, various significant correlations were identified between heavy metal concentrations measured in food, water, blood, and hair samples. However, the observed correlation coefficients were generally weak to moderate, limiting the strength of potential mechanistic interpretations. The majority of these associations involved different elements rather than the same metals, suggesting that these correlations are unlikely to reflect direct bioaccumulation. The inverse relationships identified between dietary lead and blood copper concentrations, as well as between lead levels in drinking water and blood arsenic, may reflect possible interactions in trace-element absorption or transport. Similarly, the positive correlation observed between cadmium in water and circulating copper may indicate a secondary disturbance in trace-element regulation, given that cadmium exposure has been reported to alter copper metabolism via metallothionein induction and associated regulatory feedback mechanisms (Roney and Colman 2004; Yang et al. 2024). Correlations involving hair-derived measurements warrant careful interpretation, since metal concentrations in hair can be influenced by external environmental contamination and cumulative surface deposition, in addition to internal exposure. Consequently, the moderate association observed between iron levels in water and copper concentrations in hair may be more consistent with shared environmental exposure sources rather than a biologically relevant accumulation process. Although several correlations reached statistical significance, their generally low to moderate magnitude, together with the lack of element-specific consistency and the absence of uniform patterns across the different biological matrices, indicate that these associations are unlikely to reflect clinically meaningful heavy-metal accumulation related to dietary protein sources or drinking water. Rather, the observed relationships are more consistent with indirect effects of trace-element regulation and variability in environmental exposure. Nevertheless, given the relatively small effect sizes observed in the present study, these interpretations should be considered cautiously.

In a study comparing element concentrations in dry and canned pet foods, iron, selenium, and copper levels were reported to be higher in canned foods than in dry foods, with some samples approaching or exceeding the recommended upper limits (Paulelli et al. 2018). In another similar study, toxic metal concentrations in cat foods were generally higher in dry foods, while iron concentrations were reported to be higher in canned foods (Zafalon et al. 2021). Another study reported that canned diets generally contained higher mineral concentrations, but also demonstrated a higher rate of non-compliance with FEDIAF mineral balance recommendations compared with dry diets (Davies et al. 2017). The differences observed in the elemental composition of canned and dry foods may be attributed to variations in raw materials, as well as differences in processing techniques, moisture content, packaging materials, and the analytical methods used for element analysis. Wet pet foods are typically produced by mixing ingredients, followed by filling into containers and sterilization through retort processing at high temperatures (approximately 120–130 °C), which ensures microbial safety but may also affect the physicochemical and nutritional characteristics of the final product (Hagen-Plantinga et al. 2017; Ramesh 2020).

One limitation of the present study is that potential confounding factors such as age, sex, breed, and environmental exposure were not systematically evaluated. These variables may influence heavy metal concentrations in biological matrices such as blood and hair and, therefore could partially affect the observed differences between diet groups. However, all cats included in the present study were privately owned indoor animals with no outdoor access or contact with other animals, which may have minimized potential environmental sources of metal exposure. In addition, although dietary groups were defined based on the predominant animal protein source, the possible contribution of mineral premixes and minor protein components present in the food formulations cannot be fully ruled out.

Considering the observational design of the present study, findings should be interpreted with caution and cannot be taken as definitive evidence of long-term safety. The absence of detectable accumulation within the evaluated period does not exclude the possibility of cumulative effects associated with prolonged exposure. Continued monitoring of trace and toxic elements in commercial diets, therefore, remains important for the protection of long-term feline health.

Future studies should include a larger number of pet food brands and batches from different geographical regions in order to provide a more comprehensive assessment of elemental variability in commercial diets. In addition, evaluating a broader range of diet types, including raw, freeze-dried, and home-prepared diets, may help to better understand potential differences in elemental exposure associated with feeding practices. Moreover, studies assessing biomarkers of metal exposure in biological samples such as blood, urine, hair, or tissues would provide valuable insight into the bioavailability and potential health effects of long-term dietary intake. Future studies, including well-characterized populations and considering potential confounders such as breed, age, sex, and some environmental factors in multivariable analytical models, would help to better clarify the relationship between diet type and heavy metal exposure in cats.

## Conclusions

This study assessed the concentrations of arsenic, cadmium, iron, and lead in commercial cat foods, drinking water, and biological samples, including blood and hair. For arsenic, cadmium, and iron, exposure levels associated with diet and water intake were within nationally and internationally accepted safety limits, and no indications of clinically relevant toxic accumulation were identified in the animals examined. Although copper concentrations in certain commercial cat foods were out of the recommended values, this was not accompanied by corresponding signs of copper accumulation or toxicity in blood or hair samples.

Overall, the results suggest that trace and toxic element concentrations measured in the evaluated diets and biological samples were generally within currently accepted regulatory reference limits. However, considering the observational design of the present study, these findings should be interpreted with caution and cannot be considered definitive evidence of long-term safety. The absence of detectable accumulation within the evaluated period does not exclude the possibility of cumulative effects associated with prolonged exposure. Continued monitoring of trace and toxic elements in commercial diets therefore remains important to support long-term feline health, particularly concerning copper concentrations and their regulatory upper limits.

## Data Availability

The datasets generated during and/or analysed during the current study are available from the corresponding author on reasonable request.
